# Floating Inferior Glenohumeral Ligament: A Case Report

**DOI:** 10.7759/cureus.71189

**Published:** 2024-10-10

**Authors:** Nobuharu Ishidu, Nobuyuki Yamamoto, Haruka Sato, Toshimi Aizawa, Eiji Itoi

**Affiliations:** 1 Orthopaedic Surgery, Tohoku University School of Medicine, Sendai, JPN; 2 Orthopaedic Surgery, Tohoku Rosai Hospital, Sendai, JPN

**Keywords:** bankart lesion, case report, floating inferior glenohumeral ligament, hagl, shoulder instability

## Abstract

A humeral avulsion of the glenohumeral ligament (HAGL lesion) is a relatively rare pathology seen in patients with traumatic anterior shoulder instability. A HAGL lesion combined with a Bankart lesion is called a floating inferior glenohumeral ligament (IGHL) and is rare. We report a case of floating IGHL that could not be diagnosed before surgery. A 32-year-old female presented with a fourth dislocation and underwent arthroscopic Bankart repair with the use of a flexible curved guide. During Bankart repair, the tip of the flexible drill was broken. We removed it under general anesthesia later. She had no recurrence three years after surgery and enjoyed skateboarding as a hobby.

## Introduction

Unlike a Bankart lesion, a humeral avulsion of the glenohumeral ligament (HAGL lesion) is a relatively uncommon injury in patients with traumatic anterior shoulder dislocation. Its prevalence is 2% to 9% [1-4]. A HAGL lesion combined with a Bankart lesion is called a floating inferior glenohumeral ligament (IGHL) and is rare [5-7]. We report a case with a floating IGHL that could not be diagnosed before surgery, and the tip of the flexible drill was broken when using a flexible curved guide during Bankart repair.

## Case presentation

**Figure 1:**
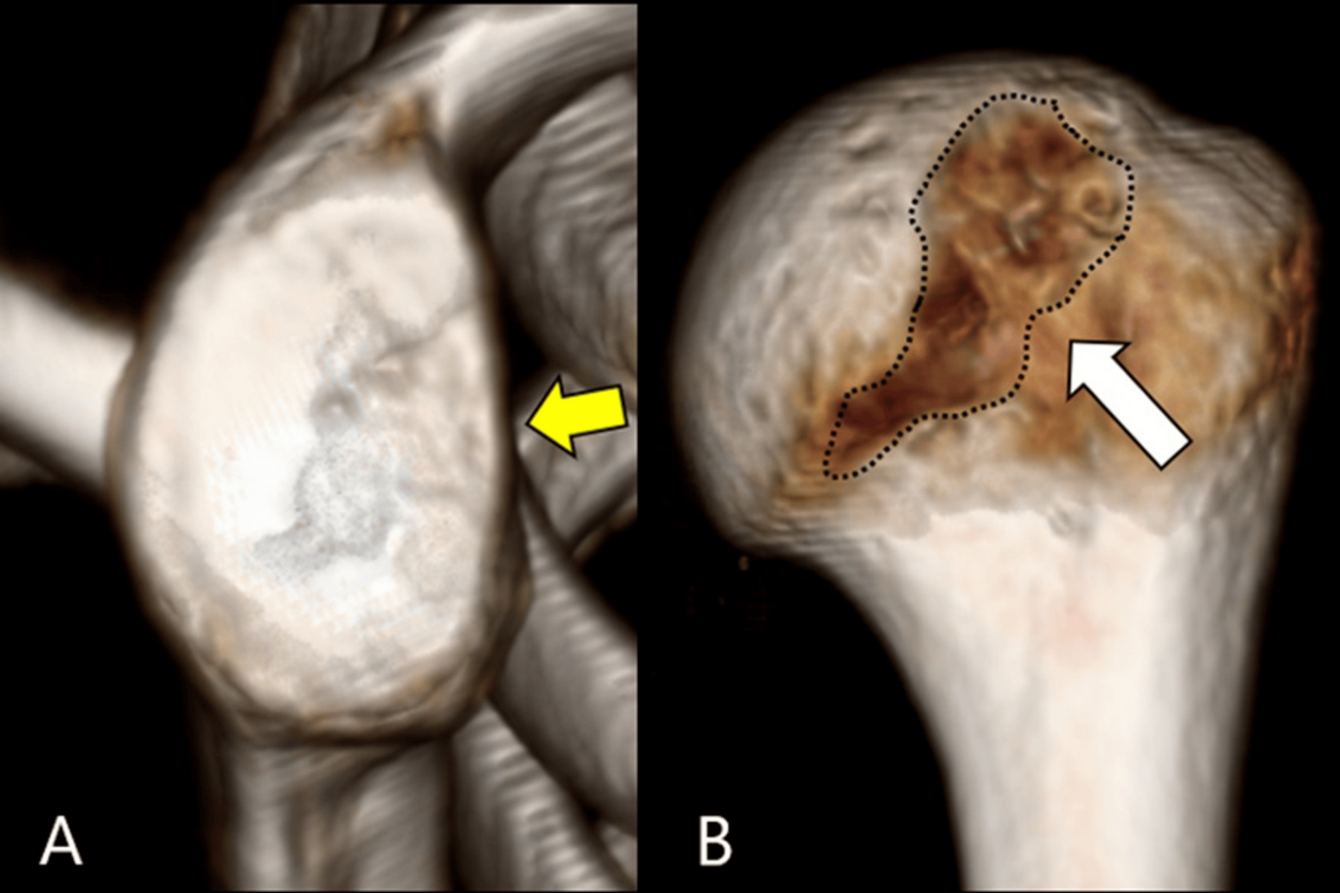
Three-dimensional CT images of the glenoid (A) and humeral head (B) The three-dimensional CT images showed a small glenoid defect (yellow arrow) and a medium-sized Hill-Sachs lesion in the posterosuperior part of the humeral head (white arrow). CT: computed tomography

**Figure 2:**
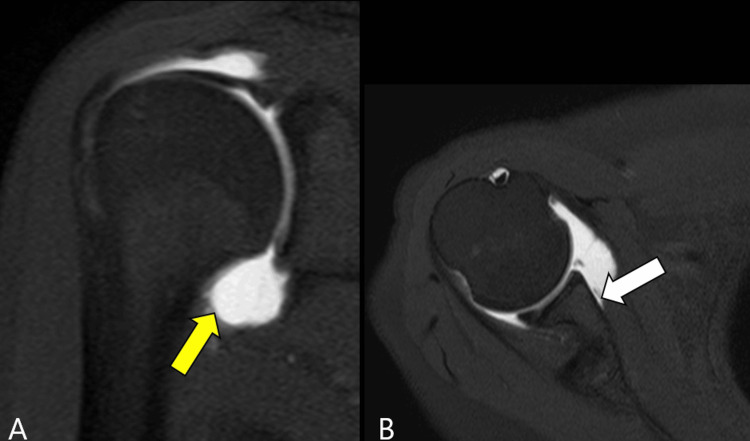
MR arthrography In the coronal images of MR arthrography, the J sign which was reported to be observed in the HAGL(yellow arrow) was not seen (A). The axial images showed avulsion of the AIGHL on the glenoid side (Bankart lesion, white arrow)(B). MR: magnetic resonance, HAGL: humeral avulsion of the glenohumeral ligament, AIGHL: anteroinferior glenohumeral ligament

A 32-year-old female presented with right shoulder instability. She suffered an initial dislocation when she fell during snowboarding and underwent manual reduction at 22 years of age. At 27 years of age, similarly, she fell while snowboarding, had dislocation twice, and underwent manual reduction. At the age of 32, she suffered the fourth dislocation while trying to catch a ball during a volleyball game and was referred to our department for further treatment. There was no past medical history. Physical examination showed that the range of motion of the right shoulder was limited due to apprehension: 120 degrees of elevation, 40 degrees of external rotation, and sacrum level of internal rotation. The apprehension test was positive. The patient had a positive sulcus sign, anterior drawer test, and posterior drawer test for shoulder joint laxity. Radiographic examination showed a Hill-Sachs lesion on the posterior aspect of the humeral head in the Stryker view. The three-dimensional CT images showed a small glenoid defect and a medium-sized Hill-Sachs lesion (on-track lesion) in the posterosuperior part of the humeral head (Figure 1). In the coronal images of MR arthrography, the J sign, which was reported to be observed in patients with a HAGL lesion, was not seen. The axial images showed avulsion of the IGHL on the glenoid side (Bankart lesion) (Figure 2).

**Figure 3:**
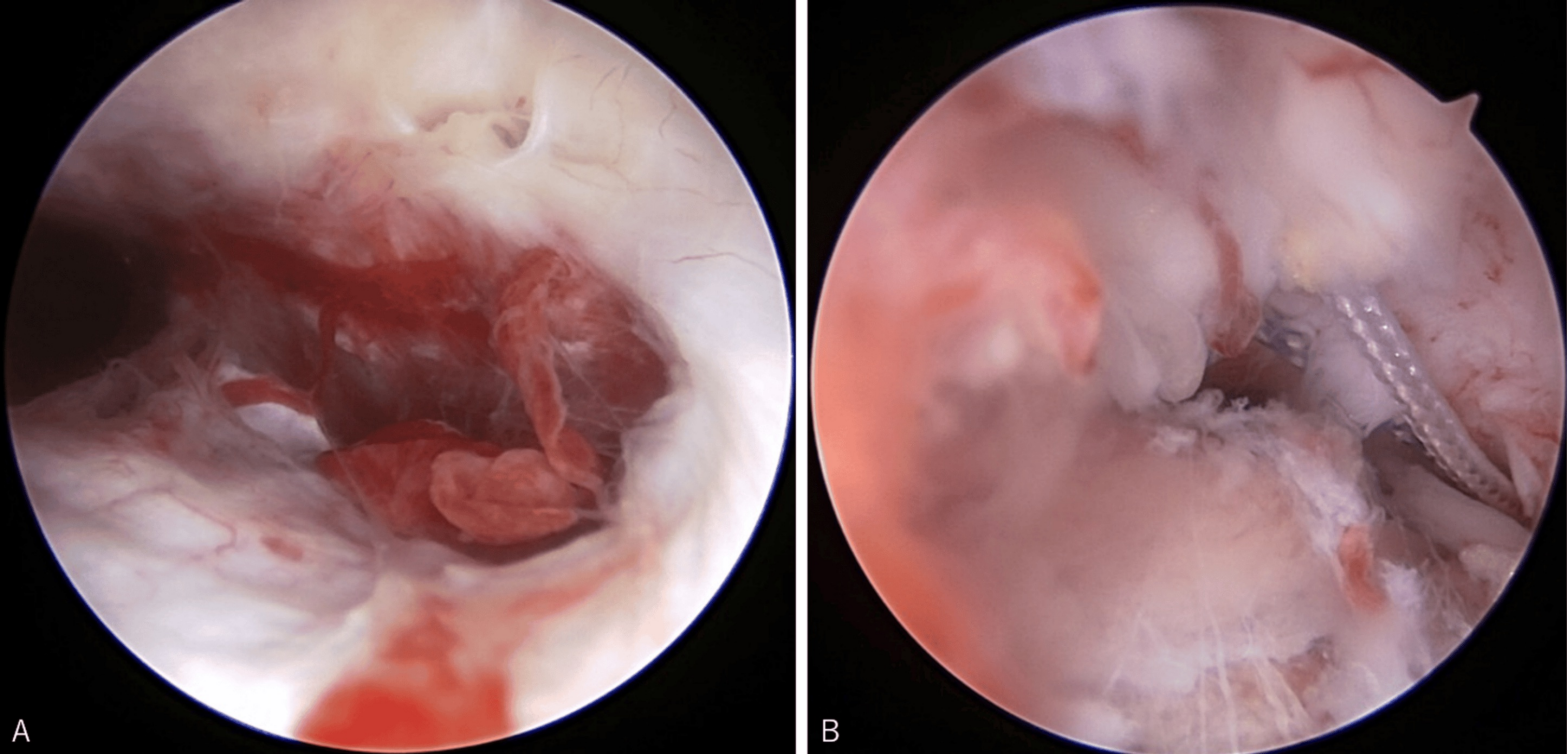
Photograph of the HAGL before and after repair Arthroscopic view from the posterior portal confirmed the HAGL lesion (A). A HAGL lesion was repaired with a simple suture technique (B). HAGL: humeral avulsion of the glenohumeral ligament

Based on the above findings, the preoperative diagnosis was recurrent shoulder dislocation with a Bankart lesion, and arthroscopic Bankart repair was planned. The surgery was performed under general anesthesia and with the patient in the beach chair position. A Bankart lesion was observed with an avulsion of the glenoid labrum from 1:00 to 4:30. There was also a HAGL lesion extending to the anterior and posterior attachment of the IGHL (Figure 3). A small glenoid bone loss was observed in the anterior part of the glenoid. There was a moderate Hill-Sachs lesion in the posterosuperior part of the humeral head.

First, an anterior portal was created at the superior border of the subscapularis tendon, followed by a five o'clock portal. One suture anchor (Gryphon, DePuy Mitek Inc., MA) with two polyethylene sutures was placed into the humeral head, and the HAGL lesion was repaired with a simple suture technique (Figure 3).

**Figure 4:**
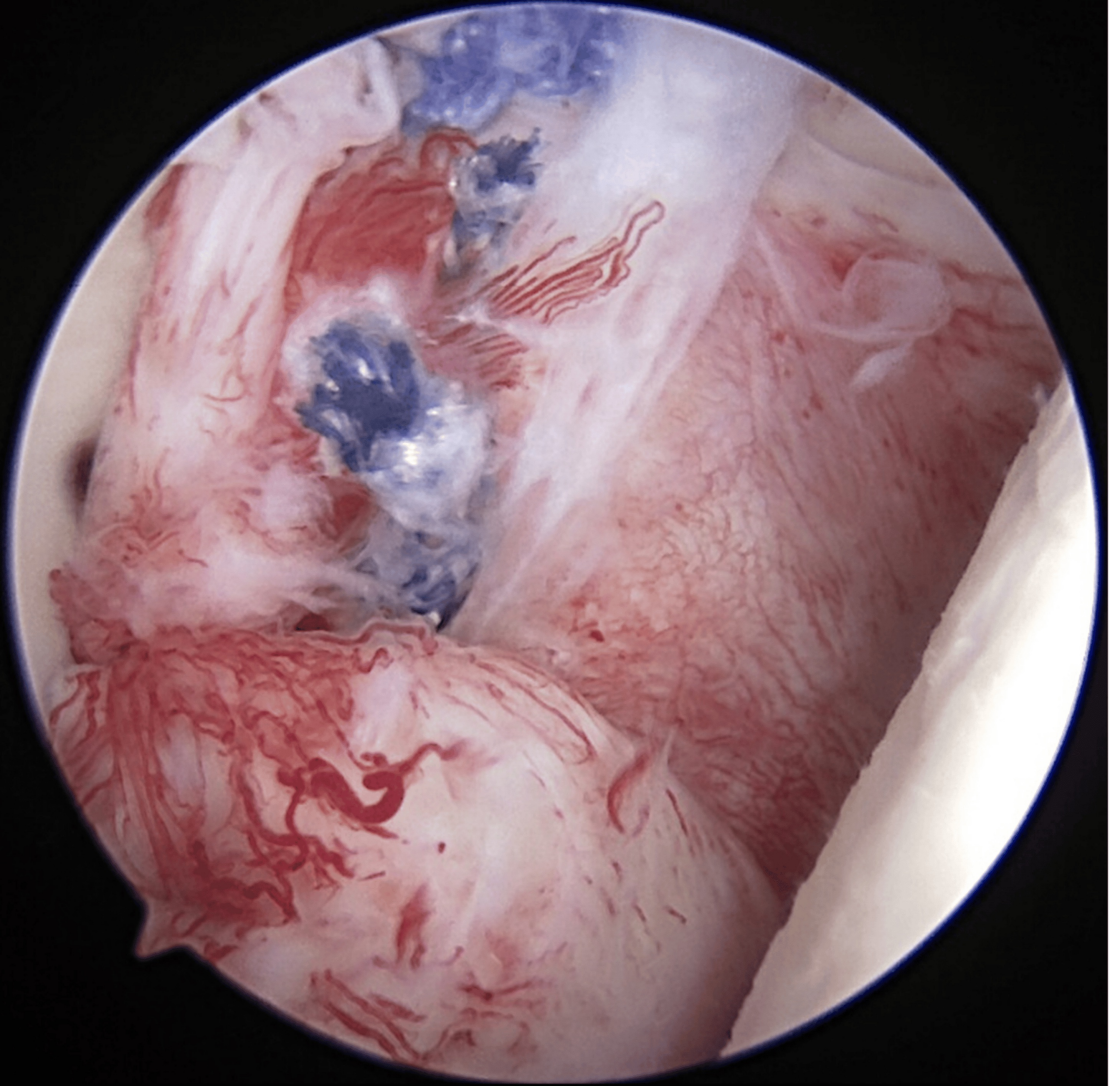
Photograph of Bankart repair A Bankart lesion was repaired by a simple suture technique. We placed four suture anchors at two, three, four, and five o’clock positions. A 2.3-mm flexible drill was inserted into the glenoid articular margin using a curved guide at the five o’clock anchor placement.

**Figure 5:**
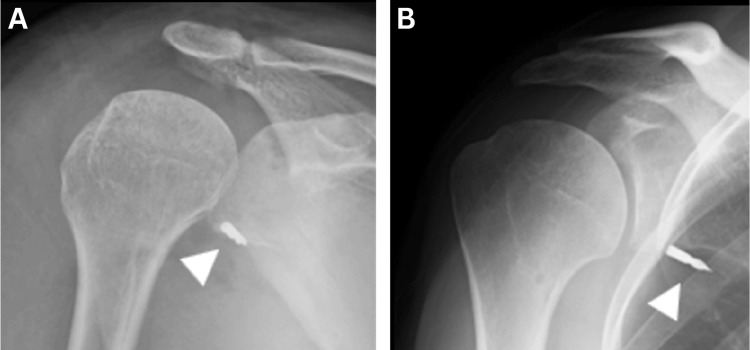
X-ray images taken soon after operation (A) and three weeks after surgery (B) The drill tip seemed to remain in the glenoid soon after surgery. However, three weeks later, the drill tip was moved.

Next, the IGHL-labrum complex was mobilized from the glenoid neck as far inferiorly as the six to seven o’clock positions in the right shoulder with the use of an elevator. Four suture anchors were placed at two, three, four, and five o’clock positions. For the five o’clock anchor placement, a 2.3-mm flexible drill was inserted into the glenoid articular margin using a curved guide (OSTEORAPTOR HA Curved 2.3 mm, Smith & Nephew Inc., London, UK). A Bankart repair was performed with a simple suture technique (Figure 4). During drilling, the drill tip was broken due to high resistance when the drill was removed. The tip of the drill was not found inside the shoulder joint, and we judged that it might have been inside the glenoid bone and would not move, and the operation was completed with the tip remaining in place (Figure 5). However, the X-ray image taken three weeks postoperatively showed the displacement of the drill tip, which was found to be inside the muscle. The patient was reoperated under general anesthesia one month after the first surgery. The location of the drill tip was confirmed under ultrasound and fluoroscopy, and a 1-cm skin incision was placed, and the drill tip was removed by using a grasper. Postoperatively, the patient was immobilized in an internal rotation brace for six weeks. Physical therapy was started the next day after surgery, focusing on isokinetic exercise until three weeks postoperatively. Three-year follow-up showed that the right shoulder was slightly limited in the range of motion with 180 degrees of elevation and 60 degrees of external rotation. She had no difficulties in her daily life and enjoyed skateboarding as a hobby. The Rowe score at the final follow-up was improved from 25 to 95 points.

## Discussion

Although more than 80% of traumatic anterior shoulder dislocation is attributed to Bankart lesion [2], other factors such as capsular tear or HAGL lesion have also been reported [2,8]. Warner and Beim first reported avulsion at both the humeral and glenoid attachments of the IGHL and termed the “floating IGHL" [5]. In the literature, the prevalence of floating IGHL is reported to be 15% to 41% among patients with a HAGL lesion [5,9,10].

Imaging has been reported to be useful in preoperative diagnosis. MR arthrography shows J-shaped changes in the axillary pouch (J sign), leakage of contrast media [3,11], disruption of the teardrop sign, and tear of the IGHL complex as the free end. The preoperative diagnosis of HAGL lesions by imaging has been reported to be 74% on MRI [12]. However, in our case, the J sign was not observed on MRI, and floating IGHL was not detected before surgery. Even if there is no J sign on the MRI preoperatively, we need to keep in mind that floating IGHL or HAGL can happen.

In our case, the drill tip was broken during drilling using a flexible drill of the curved guide system. It happened when the drill was stuck. Since the flexible part of the drill is mechanically weak, we need to be aware that there is a chance that the drill tip will be broken, especially in young patients with strong bones. When finding the broken tip, we looked for the drill tip arthroscopically, but it was not found inside the shoulder joint. From the anteroposterior view of the X-ray, we judged that it might have been inside the glenoid bone and would not move, and the operation was completed with the tip remaining in place. However, the x-ray image taken three weeks postoperatively showed the displacement of the drill tip, which was found to be inside the muscle. During surgery, we should have checked at least two directions (anteroposterior and Y-view) or confirmed whether or not there was a drill tip in the bone under fluoroscopy.

## Conclusions

We presented a 32-year-old female with a floating IGHL. A HAGL lesion was not detected on preoperative imaging. We performed a Bankart repair following the HAGL repair. During the Bankart repair, the tip of the flexible drill broke. When using the curved guide system, it is important to be aware of the mechanical weakness of the drill tip, which may lead to breakage.
